# Acknowledgement to Reviewers of *Animals* in 2014

**DOI:** 10.3390/ani5010041

**Published:** 2015-01-07

**Authors:** Animals Editorial Office

**Affiliations:** MDPI AG, Klybeckstrasse 64, CH-4057 Basel, Switzerland

The editors of *Animals* would like to express their sincere gratitude to the following reviewers for assessing manuscripts in 2014:

Adler, Steffen A.
Aloisi, Anna Maria
Anderson, Dave
Anjum, Reshma
Appleby, Michael C.
Armero Ibáñez, Eva
Atwood, Todd C.
Baker, Kate C.
Balasubramaniam, Krishna N.
Baragiola, Raúl A.
Baumans, Vera
Beisner, Brianne
Benjamin, Madonna
Bennett, Sara L.
Benson, John F.
Berg, Eric
Bergeron, Renée
Berliner, Elizabeth
Bernal, Manuel Hernando
Berry, Nick
Boronyak, Louise
Bosch, Guido
Boulouis, Henri-Jean
Bradshaw, John
Burda, Hynek
Burns, Penny
Bushby, Philip A.
Caba, Mario
Camerlink, Irene
Campbell, Madeleine
Cheng, Ken
Chowdhury, Vishwajit S.
Cox, Daniel
Cretaz von Roten, Fabienne
Croft, David
Dal Bosco, Alessandro
Dalla Villa, Paolo
De Smet, Stefaan
Dinuccio, Elio
Dombrosky, Jonathan
Dore, Kerry M.
Dowling, Seana
Draper, Chris
Duarte, Margarida
Elinson, Richard P.
Ellis, Michael
Elvidge, Chris
Faucitano, Luigi
Félix, Ananda Portela
Fidani, Cristiano
Fitzgerald, Robert
Foschi, Cristiano
Freire, Raf
Friend, Ted
Gabler, Nick
Gaughan, John
Gazzano, Angelo
Geers, Rony
Giergiczny, Marek
Goodman-Milne, Emma
Gottardo, Flaviana
Gruber, Leonhard
Hales, Kristin Eteinne
Hanlon, Alison
Hart, Benjamin
Hayakawa, Masashi
Hazel, Susan
Heerkens, Jasper
Hennessy, Michael
Herzog, Harold
Hess, Sebastian
Hilken, Gero
Hinton, Joseph W.
Holland, Richard
Humphreys, James
Hunt, Melissa
Immink, Victor M.
Jennings, Maggy
Jiang, Peihua
Jirkof, Paulin
Johnson, Nick
Johnston, Angie
Jordan, Arriaga
Kanwal, Jagmeet S.
Kephart, Kenneth B.
Koppel, Kadri
Lambert, Kim
Lambooij, Bert
Leuenberger, Fanny
Levrino, Gustavo A. Maria
Lewis, Craig
Luzi, Fabio
Lyons, Dan
Ma, Liying
Maas, Miriam
Maertens, Luc
Mandillo, Silvia
María Levrino, Gustavo A.
Marsh, Laura K
Maruyama, Soichi
Mastilović, Jasna S.
McBride, Anne
McGee, Mark
McLelland, David
Meagher, Rebecca
Mehrkam, Lindsay
Montoya Alonso, Jose Alberto
Morris, Gregory D.
Morton, David
Morzillo, Anita T.
Mugnai, Cecilia
Nanni Costa, Leonardo
Napolitano, Fabio
Němec, Pavel
O’kiely, Dr. Padraig
Olsson, Anna
Ong, Lin Kooi
Ormandy, Elisabeth H.
Orskov, Bob
Paquet, Paul
Peterson, Mandy
Porto, Simona M. C.
Rawson, Nancy
Redondo, Pedro González
Riber, Anja Brink
Rigge, Matthew
Riggio, Vincenzo A.
Robins, Dr Andrew
Rocha, Teresa
Rodger, John
Rogers, Chris W.
Rosmini, Roberto
Rossi, John
Roughan, Johnny
Ruviaro, Clandio
Sacchelli, Sandro
Schuppli, Catherine
Schwartz, Michael
Shanahan, Danielle F.
Shivik, Dr. John
Silverstein, Deborah C.
Siracusa, Carlo
Sneddon, Lynne
Sutherland, Mhairi
Taylor, Andy
Taylor, Melanie
Thiele-Bruhn, Soren
Thompson, Kirrilly
Travis, Holly J.
Trevisi, Erminio
Turner, Dennis
Tzouramani, Irene
Vaarst, Mette
Van De Weerd, Heleen
Verstegen, Martin W. A.
Villaroel, Morris
Visser, Kathalijne
Walsh, Carolyn
Weiler, Ulrike
Weiss, Jürgen
Weston, Michael A.
Wey, Tina
White, Joanne
White, Steven
Whiting, Martin
Whittaker, Alexandra
Williams, John
Zedrosser, Andreas
Zurbrigg, Kathy

